# Defect induced ferromagnetic ordering and room temperature negative magnetoresistance in MoTeP

**DOI:** 10.1038/s41598-021-88669-8

**Published:** 2021-04-27

**Authors:** Debarati Pal, Shiv Kumar, Prashant Shahi, Sambhab Dan, Abhineet Verma, Vinod K. Gangwar, Mahima Singh, Sujoy Chakravarty, Yoshiya Uwatoko, Satyen Saha, Swapnil Patil, Sandip Chatterjee

**Affiliations:** 1 Department of Physics, Indian Institute of Technology (Banaras Hindu University), Varanasi, 221005 India; 2 Hiroshima Synchrotron Radiation Center, Hiroshima University, Higashi-Hiroshima City, 739-0046 Japan; 3 Department of Physics, D.D.U. Gorakhpur University, Gorakhpur, 273009 India; 4 UGC-DAE Consortium for Scientific Research, Kalpakkam Node, Kokilamedu, 603104 India; 5 Institute for Solid State Physics, University of Tokyo, Kashiwa, Chiba 277-8581 Japan; 6 Department of Chemistry, Institute of Science (Banaras Hindu University), Varanasi, 221005 India

**Keywords:** Condensed-matter physics, Topological matter

## Abstract

The magneto-transport, magnetization and theoretical electronic-structure have been investigated on type-II Weyl semimetallic MoTeP. The ferromagnetic ordering is observed in the studied sample and it has been shown that the observed magnetic ordering is due to the defect states. It has also been demonstrated that the presence of ferromagnetic ordering in effect suppresses the magnetoresistance (MR) significantly. Interestingly, a change-over from positive to negative MR is observed at higher temperature which has been attributed to the dominance of spin scattering suppression.

## Introduction

The realization of the Weyl semimetals (WSMs) has sparked extreme research interests in condensed-matter physics community since it provides the recognition of the Weyl fermions. This topological semimetal is associated with the lack of time-reversal or inversion symmetry. The concept of WSMs can be categorized into two ways. In type-I WSMs, the linear nondegenerate band crossings lead to a point like Fermi surface (FS) when the chemical potential is adjusted to the energy of Weyl Points (WPs). The hole and electron pockets form the WPs^1,2^ in the type-II WSMs unlike the case of type-I in which band crossings produce the WPs. A finite density of states at the chemical potential is created due to overlapping of these electron and hole pockets over a range of energies^3–5^. Again the type-I WSM obeys Lorentz invariance, in contrast, type-II does not. These Weyl points are twofold degenerate and always come in pairs with opposite chirality, namely, a source and a sink of the Berry curvature^6,7^. The concept of type-II WSMs was brought forward by studying the topological properties of MoTe_2_, WTe_2_ and their alloy Mo_1−x_W_x_Te_2_^8–10^. With time many researches were devoted to 2D transition metal dichalcogenides materials (TMDs) with chemical formula MX_2_, where M is a transition metal and X is a chalcogen atom (S, Se or Te) due to their significant electronic and optoelectronic properties. Very recently, 3D TMDs WP_2_ and MoP_2_ were predicted to host four pairs of type-II Weyl points below the Fermi energy with a unique feature of having same chirality for the nearest WPs^11^.

In addition, WSMs are in general renowned for their negative longitudinal magnetoresistance (NLMR) induced by chiral anomaly^12,13^, which refers to the non-conservation of chiral charge around the Weyl nodes when applied electric and magnetic fields are non-orthogonal (E.B ≠ 0). The experimental measurement of NLMR is very sensitive, and especially for type-II WSMs, the NLMR can only be observed along specific crystalline directions and in samples with appropriate chemical potential^14,15^. In type-I Weyl semimetals, the chiral anomaly always appears regardless of the direction of the applied magnetic fields. Thus the observation of positive longitudinal magnetoresistance under applied magnetic field perpendicular to electric field is strong evidence to distinguish type-II WSMs from type-I WSMs. To best of our knowledge, so far, the NLMR has not been reported for MoTe_2_^16^.

Furthermore, there are many reports on the spin–orbit coupling and the interesting consequences of electrical and optical properties in these systems. However, there are very limited, and mostly theoretical studies on intrinsic magnetism based on monolayer structure calculation^17–20^. Theoretical and experimental work shows that in the absence of crystalline imperfections, the Mo-based TMDs are nonmagnetic^21–23^. Therefore, by adding defects one may induce magnetism into these materials and this ability can open up a host of new opportunities for spintronic applications. In this report we have investigated for the first time the type-II Weyl semimetallic MoTeP. Our study indicates that it is a defect induced magnetic Weyl semimetallic material.

## Methods

### Sample preparation

Single crystals of MoTeP were grown by the chemical vapor transport method^24,25^ in 3 steps. In the first step polycrystal of MoTeP was synthesized by heating stoichiometric amounts of Mo, Te and P powders at 800 °C for 24 h. This MoTeP polycrystal was again sealed in an evacuated quartz ampoule with iodine as a transport agent. The ampoule is put in the two-zone furnace with a temperature gradient 1050 °C (source) to 950 °C (sink) for two weeks and then quenched in ice-cold water. This fast cooling process yields 1 Tʹ-phase of MoTeP single crystal.

### Structural characterization

Figure S1 of supplementary shows the room temperature X-ray diffraction (XRD) pattern of the MoTeP material. We have performed Le-Bail refinement of the powder XRD data (CuK_α_ radiation) of MoTeP single crystal to obtain the lattice parameters. The extracted lattice parameters are *a* = 3.3759(52) Å*, b* = 6.2907(93) Å and *c* = 13.8629(28) Å whereas the calculated α, β and γ values are 90°, 94.82° and 90°. The obtained lattice parameters are consistent with 1 Tʹ (P2_1_/*m*) family of compounds^26^.

### Material characterization

Magneto-transport properties measurements were performed in a Quantum Design Physical Properties Measurement System (PPMS) using a conventional four-probe configuration. The measurements were performed in a standard Hall bar geometry i.e. electrical current was applied along the b axis, and magnetic field perpendicular to the b axis. Magnetic measurements (both temperature and magnetic field dependent) were performed using quantum design SQUID magnetic properties measurement system (MPMS). Temperature dependent Raman study was executed by Horiba LabRam HR evolution spectrometer. The sample was irradiated with 633 nm He–Ne laser. The sample was cooled from 300 to 190 K by liquid nitrogen when taking Raman measurements at different temperatures.

### The density functional theory (DFT) calculations

We performed DFT calculations using ABINIT package^27^ with a projector-augmented-wave (PAW) method. We adopted generalized gradient approximation (GGA) as exchange–correlation proposed by Perdew-Burke-Ernzerhof (PBE). All atoms of MoTeP were fully relaxed with conjugate-gradient algorithm until a force is less than 0.01 eV Å^−1^ and the energy convergence criteria was put to 10^–6^ eV. The electronic calculations were performed using Γ-centred K-mesh of 15 × 9 × 5 with a plane-wave energy cutoff of 19.1096 Ha (520 eV), spin orbit coupling (SOC) were included in the calculations. We used optimized lattice parameters (a = 3.3418(56) Å, b = 6.0887(71)Å and c = 13.3433(95)Å for the theoretical calculation of MoTeP. For the spin-polarised DOS calculations vacancy and antisite defects were created inside the crystal structure. Additionally, we checked GGA + U with U = 0.0367493 Ha (1 eV), however inclusion of Coulomb interaction U does not affect our results. The output files (.agr) were analysed in XMGRACE^28^ software.

## Results and discussion

### Zero field resistivity behavior

**Figure 1:**
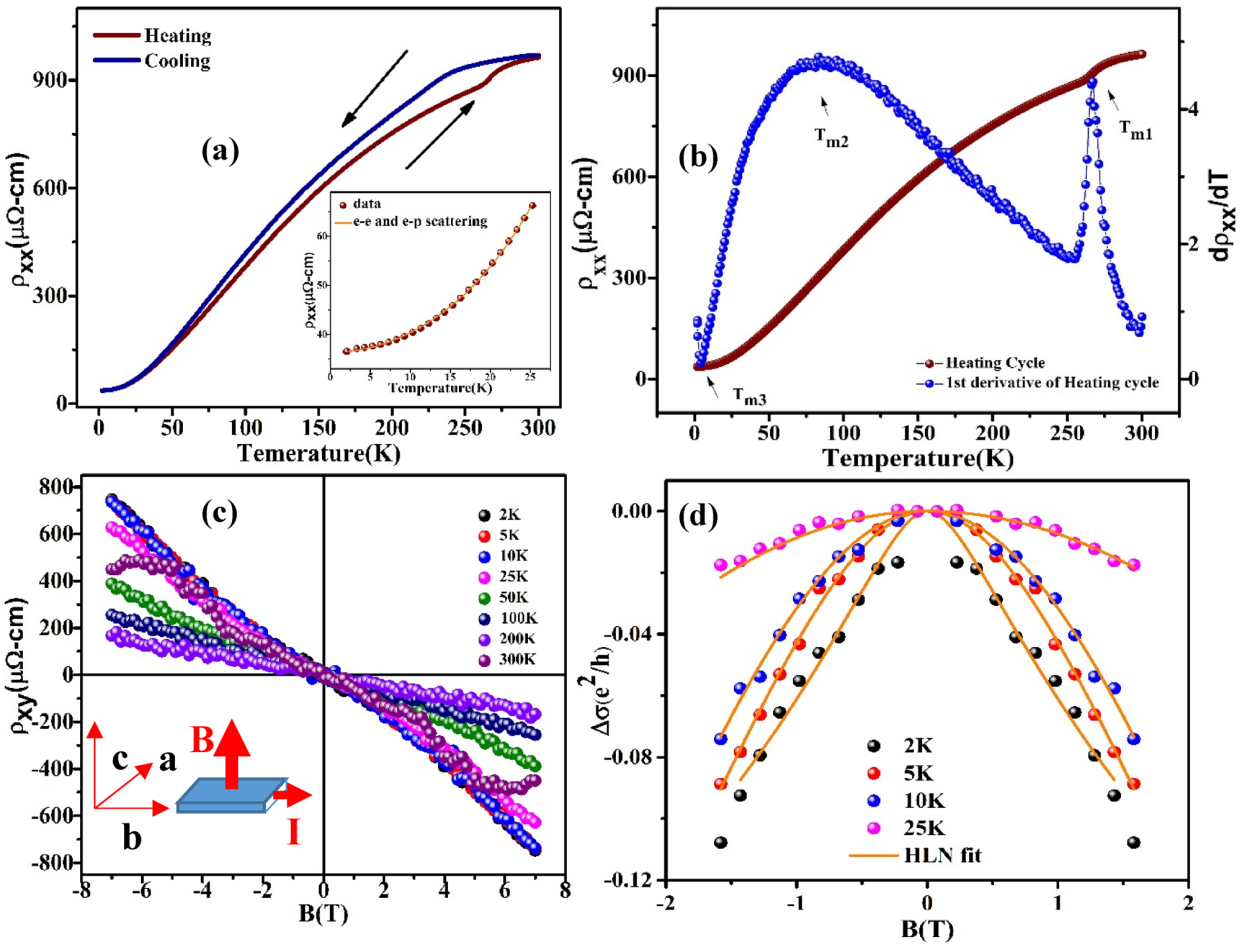
(**a**) Thermal hysteresis of temperature dependent resistivity of 1 Tʹ MoTeP, inset: low temperature resistivity data, orange solid line is a fit of electron–electron and electron–phonon scattering terms ρ(T) = ρ_0_ + aT^2^ + bT^5^, (**b**) temperature evolution of ρxx and dρ_xx_/dT at zero magnetic field, (**c**) Field dependence of Hall data ρ_xy_ at various temperatures, inset: measurement geometry, (**d**) low-field low-temperature conductivity data, solid orange line shows HLN fitting to Δσ(e^2^/h) at various temperatures.

The temperature evolution of the resistivity ρ_xx_ shows metallic character throughout the measured temperature range as shown in Fig. 1a. The longitudinal resistivity ρ_xx_ indicating dominant electron–electron and electron–phonon scattering at low temperature as depicted in Fig. 1a inset. MoTeP shows resistivity 36.55 μΩ-cm at 2 K and this reaches to 963.47 μΩ-cm at 300 K. Thus the residual resistivity ratios (RRRs) = ρ (300 K)/ρ (2 K) = 26.41 suggesting good crystalline quality of the sample. This value of RRR is consistent with several topological semimetals^29–31^.

The temperature dependence of the resistivity at low temperature can be accounted for by the usual combination of Fermi liquid and electron–phonon scattering mechanisms by fitting to ${\rho}_{xx}(T)={\rho}_{0}+a{T}^{2}+b{T}^{5}$, where ρ_0_ = ρ(T = 0 K) with a and b being fitted parameters (Fig. 1a inset). Three temperature regions T_m1_, T_m2_, T_m3_ are marked with arrows in Fig. 1b. The observed hysteresis around T_m1_ ~ 250 K between the warm-up and the cool-down curves (Fig. 1a) is ascribed to the structural phase transition from the 1 Tʹ to the T_d_ structure which is consistent with MoTe_2_^32,33^. This structural phase transition is confirmed by temperature dependent Raman spectroscopy (supplementary). The coexistence of 1 Tʹ and T_d_ phase is the possible reason behind the hysteresis over a long range of temperature. This type of hysteresis behavior is common in MoTe_2_^32^. The dρ_xx_(T)/dT curve exhibited a broad peak around the temperature ~ 75 K (marked as T_m2_), which suggests the possible change in the electronic structure of T_d_-MoTeP. This is also consistent with the reports on MoTe_2_^24,34–37^. However, the temperature (T_m2_) for MoTe_2_ is ~ 50 K. Temperature dependent mass anisotropy was also reported around T_m2_ in MoTe_2_ by Chen et al.^34^. We identified the temperature as T_m3_ where 1st derivative of ρ_xx_ becomes minimum which is also known as the turn on temperature^34^. Therefore, the observed transport behavior in MoTeP is consistent with the Weyl semimetal MoTe_2_^32^. In T_d-_ phase inversion symmetry is broken which is the necessary condition for a material to have Weyl semimetal phase^8,38,39^ In the present investigation, the existence of T_d_-phase in MoTeP is a possibility of this material to be Weyl semimetallic. Furthermore, turn on behavior is commonly attributed to field induced metal insulator transition and is well familiar among extremely large MR (XMR)^32,37,40–42^. Importantly, Q. L. Pei and his group^37^ suggested the electronic structure change (near 50 K) as the necessary condition for the presence of the turn-on phenomenon in WTe_2_ and T_d_-MoTe_2_.

### Hall effect study

Figure 1c displays the magnetic field dependence of Hall resistivity ρ_xy_. From the Hall resistivity behaviour it is clear that the conduction carriers are dominated by electrons which is consistent to that of other Weyl semimetals^43,44^. However, the ρ_xy_(B) shows non-linear behavior and with increase of temperature the deviation from linearity of ρ_xy_(B) increases and finally at 300 K it shows unusual non-linearity (S-shaped) at high field. This nonlinear ρ_xy_(B) implies the existence of both electrons and holes. The S-shaped nonlinearity is similar to that of topological single crystal system Bi_1-x_Sb_x_^45^. In order to determine carrier mobility and carrier density for both type of charge carriers we executed two-carrier model fit with our σ_xy_ and σ_xx_ data, where the field dependence of the conductivity tensor is given by

1 $$\varvec{\sigma}_{{\varvec{x}}{\varvec{y}}}=\left[{{\varvec{n}}}_{{\varvec{e}}}{{{{\upmu}}}_{{\varvec{e}}}}^{2}\frac{1}{1+\boldsymbol{ }{\left({{{\upmu}}}_{{\varvec{e}}\boldsymbol{ }}{\varvec{B}}\right)}^{2}}-{{\varvec{n}}}_{{\varvec{h}}}{{{{\upmu}}}_{{\varvec{h}}}}^{2}\frac{1}{1+({{{{\upmu}}}_{{\varvec{h}}}{\varvec{B}})}^{2}}\right]\mathbf{e}\mathbf{B}$$

where Hall conductivity

2 $$\varvec{\sigma}_{{\varvec{x}}{\varvec{y}}}=-\frac{{\rho}_{{\varvec{x}}{\varvec{y}}}}{{{{\rho}_{{\varvec{x}}{\varvec{x}}}}^{2}+{\rho}_{{\varvec{x}}{\varvec{y}}}}^{2}}$$

**Figure 2:**
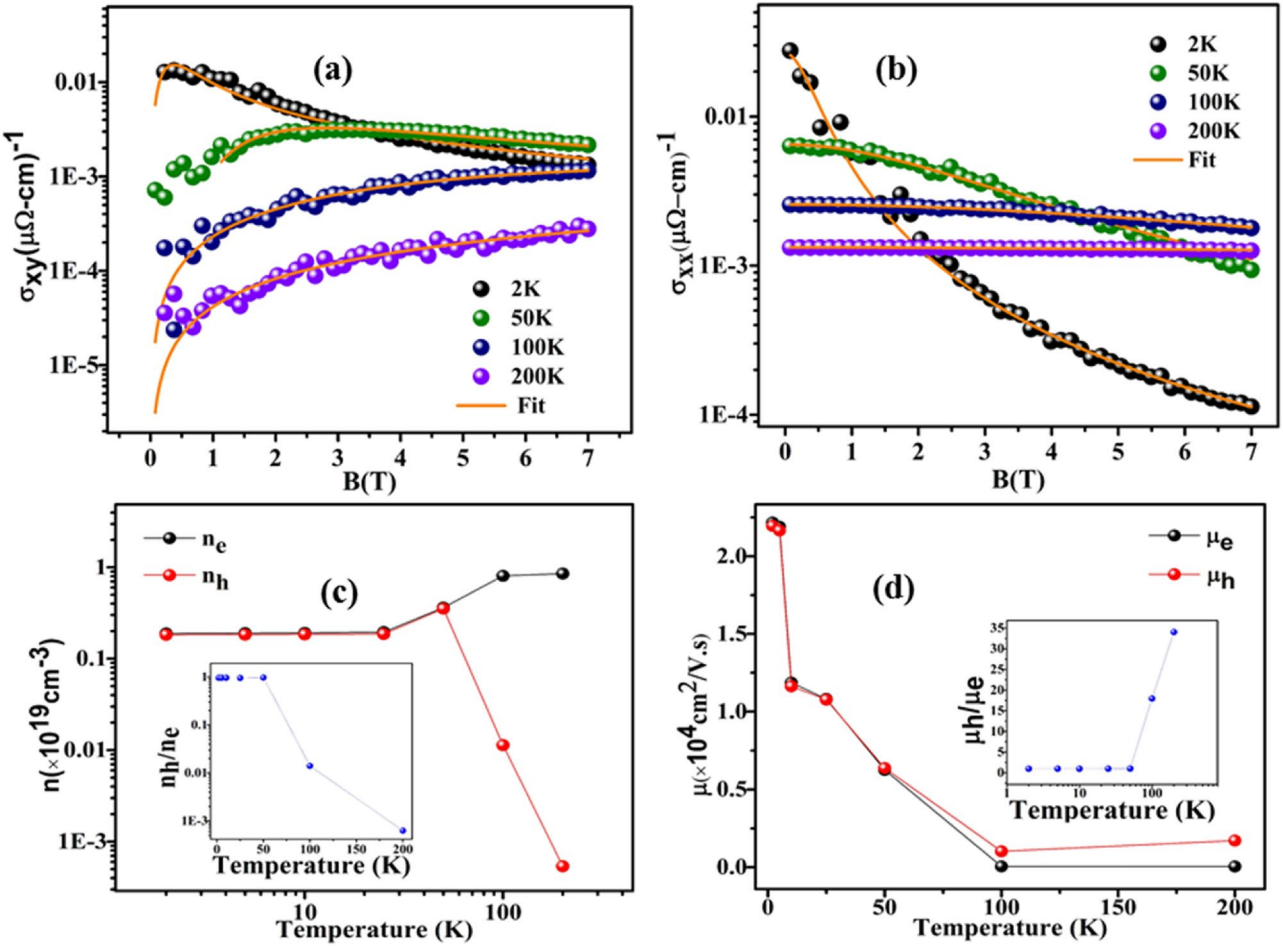
(**a**,**b**) Field dependence of Hall conduvtivity σ_xy_ and longitudinal conductivity σ_xx_ at 2,50,100 and 200 K, orange solid lines are their respective two-carrier model fit with Eqs. (1) and (3), (**c**) density of electrons n_e_ (black circle) and density of hole n_h_( red circles) as a function of temperature extracted from σ_xy_, inset: ratio of n_h_ and n_e_ as a function of temperature, (**d**) electron mobility μ_e_ (black circle) and hole mobility μ_h_ ( red circles) and their ratio μ_h_/μ_e,_ inset: as a function of temperature.

Here, n_e_(n_h_) and μ_e_(μ_h_) are electrons (holes) carrier densities and mobilities, respectively and σ_xy_ is the Hall conductivity. Figure 2a illustrates the temperature dependence of the Hall conductivity and their respective two-band model fit. The fitting of Eq. (1) yields electron and hole densities 0.188 × 10^19^ and 0.182 × 10^19^ cm^−3^ respectively at 2 K. The electron and hole mobilities are 2.216 × 10^4^ and 2.196 × 10^4^ cm^2^/V s. The extracted parameters n_e_, n_h_, μ_e_, μ_h_ and their temperature evolution along with a comparative result of n_h_/n_e_ and μ_h_/μ_e_ is also shown in Fig. 2c,d. This values show that the magnetotransport properties in MoTeP is primarily influenced by electron type charge carriers and a near perfect electron–hole compensation scenario is present in this system at low temperature. These values are comparable to many Dirac Cd_3_As_2_, ZrTe_5_^46,47^ and other semimetallic MoTe_2_, WTe_2_, LaSbTe, VAl_3_^24,48–50^ systems. However, our hole mobility is two order less than WP_2_^42^. The carrier density is almost constant from 2 to 15 K. However, the mobility of both type of carriers decreases with increase in temperature. Above 50 K hole mobility and electron density increases rapidly and there is a change in the temperature dependence of the electron and hole density and/or mobility. The electron and hole density extracted at room temperature n_e_ ~ 0.177 × 10^19^ cm^−3^, n_h_ ~ 0.173 × 10^19^ cm^−3^ and their mobilities are μ_e_ ~ 0.196 × 10^4^ cm^2^/V s and μ_h_ ~ 0.198 × 10^4^ cm^2^/V s respectively. Furthermore, it is observed that the amplitude of the nonlinear S-shaped becomes flat with increasing n_e_. Also, hole plays dominant contribution at this temperature with an increase in μ_h_. In order to assess the accuracy of the parameters obtained for charge density and mobility we further fitted our σ_xx_ (Eq. 3) data using

3 $$\varvec{\sigma}_{{\varvec{x}}{\varvec{x}}}=\left[\frac{{{\varvec{n}}}_{{\varvec{e}}\boldsymbol{ }}{\varvec{e}}\boldsymbol{ }{{{\upmu}}}_{{\varvec{e}}}}{1+{\left({{{\upmu}}}_{{\varvec{e}}\boldsymbol{ }}{\varvec{B}}\right)}^{2}}+\frac{{{\varvec{n}}}_{{\varvec{h}}\boldsymbol{ }}{\varvec{e}\boldsymbol{ }}{{{\upmu}}}_{{\varvec{h}}}}{1+{\left({{{\upmu}}}_{{\varvec{h}}}{\varvec{B}}\right)}^{2}}\right]$$

where longitudinal conductivity

4 $$\varvec{\sigma}_{{\varvec{x}}{\varvec{x}}}=\boldsymbol{ }\frac{{\rho}_{{\varvec{x}}{\varvec{x}}}}{{{\rho}_{{\varvec{x}}{\varvec{x}}}}^{2}+{{\rho}_{{\varvec{x}}{\varvec{y}}}}^{2}}$$

σ_xx_ is longitudinal resistivity (Fig. 2b) in transverse magnetic field and current configuration. The obtained electron density n_e_ ~ 0.188 × 10^19^ and hole density n_h_ ~ 0.182 × 10^19^ cm^−3^ and the extracted electron mobility μ_e_ ~ 2.218 × 10^4^ cm^2^/V s and hole mobility μ_h_ ~ 2.164 × 10^4^ cm^2^/V s at 2 K. The hole density increases beyond 50 K whereas and electron–hole density becomes comparable at low temperature. As evident from the Fig. 2c n_e_ increases with temperature whereas n_h_ decreases above 50 K. This is similar to MoTe_2_^34^. In addition, mobility of both type of carriers decreases with increasing temperature. The ratio n_h_/n_e_ decrease above 50 K. Above a certain temperature hole mobility becomes larger than electron mobility. Finally, at room temperature both the carriers are taking part in the transport. This might be the reason of S-shaped nature of Hall data at 300 K.

It is worthwhile to mention that the large carrier mobility is decisive to the XMR effect in Weyl semimetal^24^. In the present case, in T_d_-MoTeP we also observe (discussed above) the large carrier mobility. However, such colossal XMR effect is absent in the present system due to dominating ferromagnetic ordering effect (discussed below). The decreased mobility at high temperature can be expected as electron–phonon scattering is dramatically increased at high temperature.

### Magnetization behavior

**Figure 3:**
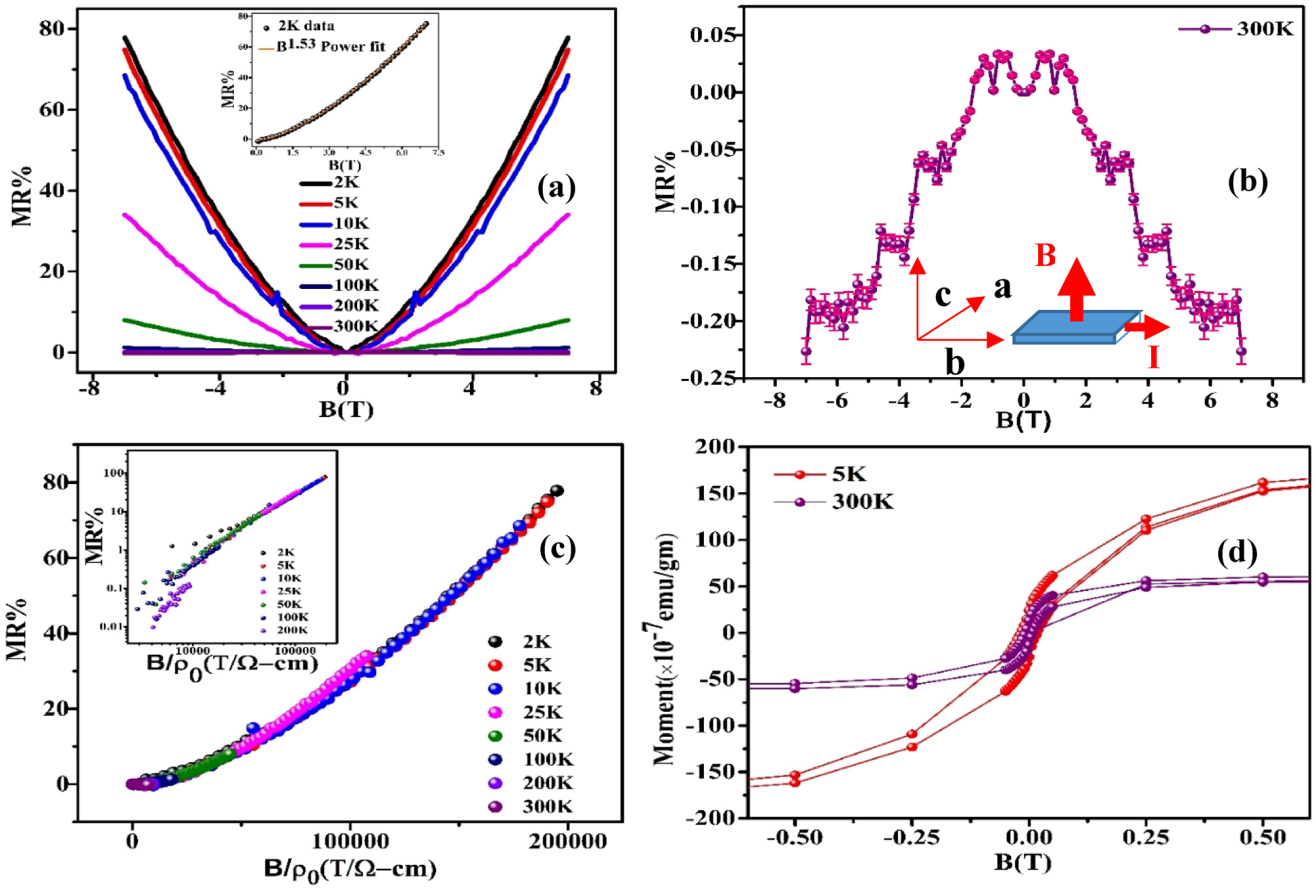
(**a**) Magnetic field dependent MR% at various temperatures ranging from 2 to 300 K, inset shows 2 K MR% with a power law fit (orange solid line), (**b**) clear pictorial view of positive MR at low field and negative MR at high field for 300 K data, inset: measurement geometry, (**c**) Kohler’s scaling of MR% as a function of B/ρ_0_ up to 300 K, inset: Kohler plot on logarithmic scale up to 200 K, (**d**) M-B magnetic hysteresis curve taken at 5 K and 300 K.

In order to find out the magnetic ordering we have also measured the magnetization behavior in MoTeP. Interestingly, we have found the presence of magnetic hysteresis in M(B) (Fig. 3d) indicating the ferromagnetic ordering in this compound. The magnitude of the loop decreases with increasing temperature. The estimated coercive fields are 278 and 195 Oe at 5 K and 300 K, respectively. This type of long range ordering is also reported in the pioneering work by Guguchia^51^ for the compound 2H-MoTe_2_ and MoSe_2_. Such ferromagnetism was induced by defects like metal vacancies and chalcogen-metal antisites disorder. The Mo vacancy can also induce spin polarization with large magnetic moments^22^. Presence of vacancy (V_Mo_, V_Te_, V_P_) and antisite defects (Te_P_, Mo_Te_ Te_Mo_) has been considered as most common point-defects in the 2D materials like MoTe_2_^17^.

### Effect of magnetic field on longitudinal resistivity

The longitudinal resistivity ρ_xx_ measured under perpendicular current and magnetic field configuration. This ρ_xx_ follows a nearly quadratic dependence on magnetic field indicating no sign of saturation.

Magnetoresistance [MR = {ρ_xx_(7T) − ρ_xx_(0T)}/ρ_xx_(0T), ρ_xx_ is the longitudinal resistivity] reaches 77.831% at 2 K. This MR value is relatively small compared to the other reports on XMR family of compounds like MoTe_2_, WTe_2_, MoP_2_ and WP_2_^34,43,44,52^. Moreover, electron–hole compensation and electronic changes near 60 K (50 K) was considered as the driving force behind the XMR in MoTe_2_ (WTe_2_)^44,53^. Particular orbital texture on the electron pocket was also proposed as a possible reason behind the XMR in MoTe_2_^34^. The MR% value is gradually suppressed with increasing temperature. The MR% decreases to 0.113% at about 200 K (Fig. 3b). A clear view of 300 K MR% data with error bar is represented in Fig. 3b. The positive MR weakened as the temperature is increased and finally the MR displayed mixed behavior from positive in low fields to negative in high fields when the temperature increased to 300 K. However, all the data in Fig. 3c can be scaled onto a single line when MR plotted as a function of B/ρ_0_. However, deviation from the single line is observed above 50 K when plotted on log–log scale (inset of 3c). Departure from the scaling signify the existence of both type of charge carrier in the system supporting the Hall data. Multiband effect with different scattering time was also considered as a reason behind the breakdown of Kohler’s rule in MoP_2_^54^. The dominance of phonon scattering at high temperature might also be the reason of this deviation^55^. However, according to semi-classical two-band theory, the validity of Kohler’s rule with MR ∝ (B/ρ_0_)^2^ suggests an XMR or perfect electron–hole compensated system. Violation of Kohler’s rule is common in XMR materials, such as LaBi, TaAs, TaAs_2_, NbAs_2_, NbSb_2_, and LaSbTe^49,56–59^. Our trial to fit the Kohler’s law (${\varvec{M}}{\varvec{R}}= {\varvec{c}} \left({\frac{{\varvec{B}}}{{\rho}_{0}}}\right)^{{\varvec{m}}}$) yields c = 25 (μΩ-cm/T)^1.65^ and m = 1.65. The value of m ~ 1.65 is away from a perfect electron–hole compensation (m ~ 2) situation. The deviation in Kohler’s scaling is also in line with our nonlinear Hall data and multiband transport above 50 K. We performed a power law (Fig. 3a inset) with MR% data at 2 K and fitting yields n = 1.53 which shows a subquadratic field dependence^60,61^. MR ~ B^n^ where n is predicted to be 2 for semimetals with perfect electron–hole compensation. Our fitted n ~ 1.53 value also conveys the results of electron dominating transport at low temperature in accordance with the Hall data.

Nevertheless, the chiral anomaly induced negative LMR and positive transverse magnetoresistance (TMR) as a result of Lorentz force is fingerprint of the type-II Weyl semimetals^37,42^. In our case MoTeP also shows positive MR up to 200 K due to Lorentz force in perpendicular current and magnetic field configuration. The MR near B = 0 is also positive at 300 K. On the other hand, under the application of external magnetic field the decrease in resistivity with increasing temperature is systematic in T_d_-MoTeP up to 200 K, as expected in semimetallic systems. The 2 K MR curve shows a small cusp-like feature at low field suggesting the presence of the weak anti-localization (WAL) effect. The conductivity change Δσ = Δσ(B) − Δσ(0) arising from the quantum interference effects is explained by the Hikami-Larkin-Nagaoka (HLN) theory^62^:

5 $$\Delta \sigma ={\varvec{\sigma}}\left({\varvec{B}}\right)-{\varvec{\sigma}}\left(0\right)={\varvec{A}}\left[{\varvec{\psi}}\left(\frac{1}{2}+\frac{{\varvec{h}}}{8{\varvec{\pi}}{\varvec{e}}{\varvec{B}}{{\varvec{L}}}_{\boldsymbol{\varphi }}^{2}}\right)-{\varvec{l}}{\varvec{n}}\left(\frac{{\varvec{h}}}{8{\varvec{\pi}}{\varvec{e}}{\varvec{B}}{{\varvec{L}}}_{\boldsymbol{\varphi }}^{2}}\right)\right],$$

where ${\varvec{A}}=\frac{\boldsymbol{\alpha }{{\varvec{e}}}^{2}}{{\varvec{\pi}}{\varvec{h}}}$ and α is a constant equal to 1 or − 1/2 for weak localization or anti-localization respectively. *ψ* is digamma function, *L*_*φ*_ is the phase coherence length. We have calculated magnetoconductance per conduction channel Δσ/Z* (Z* is no. of conduction layers). One 2D layer corresponds to e^2^/h conductance and that is equal to 2 QL thickness i.e. 2 nm. Therefore, Z* would be equal to t/2 nm^63^ where t is thickness of the sample. The 2 K data of Δσ (e^2^/h) shows a small phase coherence length, L_ϕ_, of about 29.98 nm indicating the weak WAL effect (Fig. 1d). The results are in accordance with previous WAL data in these family of compounds^64^. The calculated α value − 0.449 confirms the WAL effect in the system. This quantum interference effect is significator of metallic state in the strong SOC system. This also indicates an enhanced spin scattering at this temperature. In addition to that, a negative α value confirms the WAL effect at low T and B range. At higher temperature WAL suppressed due to enhanced spin dependent scattering. The fitted values of L_Φ_ are 29.98 nm (A = − 0.14 Ω^−1^), 14.21 nm (A = − 0.82 Ω^−1^), 10.28 nm (A = − 1.96 Ω^−1^) and 5.38 nm (A = − 6.73 Ω^−1^) at 2, 5, 10 and 25 K respectively. The calculated value of α is compared with other reports on TMDs materials in Table 1.

**Table 1:** The values of α extracted from HLN fitting.

Temperature	α	Temperature (K)
MoTe_2_^65^	− 0.41 to − 0.51	< 2.5 K
MoTe_2_^66^	− 0.8	1.5 K
WTe_2_^67^	< < 1	2 K
MoSe_2_^68^	0.56, 0.49	–
MoTeP (present work)	− 0.449	2 K

Most interestingly, we have found negative magnetoresistance near room-temperature at high field. In a topological system few possibilities are there behind negative MR like—(a) chiral anomaly in Weyl semimetals, (b) current jetting effects, (c) weak localization effect, (d) ferromagnetism in the sample and (e) field induced magnetic impurity scattering. Observation of the chiral-anomaly induced negative MR requires the applied magnetic field to be parallel to the electric field^47,69–71^, which is not our case. An inhomogeneous distribution of the current flowing inside the sample can give rise to negative MR effect and current jetting^72^ shows strong dependence on sample geometry/size. However, in our case observed MR is systematically decreased to a negative value and is not observed in the whole range. Furthermore, this effect requires strong preference of the current to flow in the direction of the magnetic field^73^. This also rules out the possibility of current jetting effect in the present investigation. On the other hand, weak localization effects^74,75^ can cause negative magnetoresistance in impurity induced semimetals and semiconductors at low field. When two electron waves interfere constructively while travelling from opposite direction along a closed path, they scatter off by the impurity and leads to an increase in magneto conductivity. In our case, the magnetoconductivity decreases with increase in field, below 1 T indicating the effect of WAL. But at higher field at 300 K the observed negative MR cannot be due to the WAL.

It signifies that the magnetism plays an important role in the transition between positive and negative MR for 1 Tʹ MoTeP. The low-field positive MR becomes parabolic like at 300 K. Under the application of external magnetic field electronic scattering rate from local moments and impurities is suppressed resulting in increase in transport lifetime that results in a negative magnetoresistance. However, considering such effect for a particular field range is not appropriate rather we can expect the not so large overall MR effect is due to the ferromagnetism in the sample^73^. Therefore, ferromagnetic ordering throughout the whole temperature range of measurement is the origin of low MR in the system. In fact, ferromagnetic ordering suppresses the scattering which in effect decreases the MR. Moreover, in Weyl semimetal the XMR decreases with increase of temperature and in the present case at 300 K the ferromagnetic ordering dominates over the scattering effect leading the negative MR.

**Figure 4:**
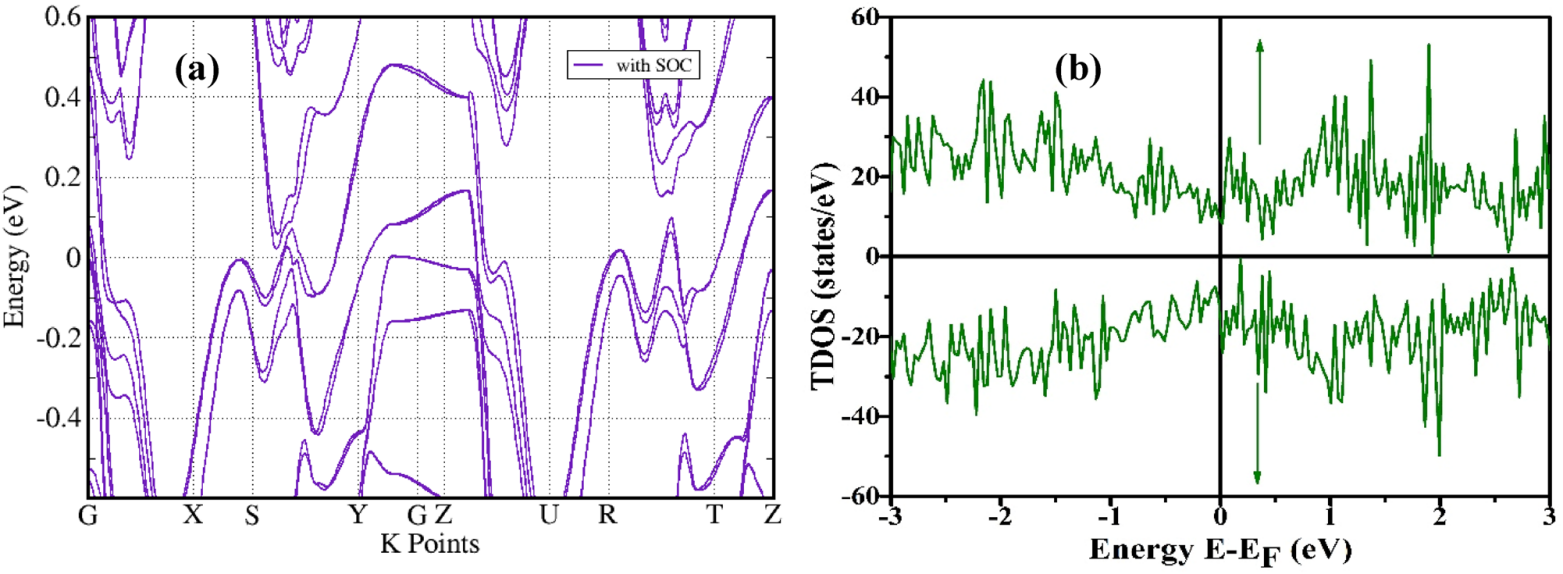
(**a**) Bulk band structure of T_d_-MoTeP with inclusion of SOC, (**b**) Spin-resolved Total DOS for defect induced MoTeP.

In order to further determine the origin of FM ordering we have performed the spin polarized DOS calculation (Fig. 4b). It is observed that MoTeP exhibits semimetallic features in bandstructure (Fig. 4a) similar to parent MoTe_2_^76^. We speculate that the inclusion of SOC splits the hole and electron bands in two sets of hole and electron pockets with slightly different sizes. The hole bands are comparatively flatter than the electron bands in MoTeP indicating holes possess greater effective mass and smaller mobility than the electrons. This corroborates well with our experimental results. The TOTAL DOS and difference DOS between spin up and spin down contribution are illustrated in Fig. 4b and S7 of supplementary. The asymmetric total DOS near the Fermi level associated with ferromagnetic behavior of the material. Furthermore, it is observed that Mo-*d* states, Te-*p* and P-*p* have the dominant contribution in their fat band calculations as shown in S5–S7 of supplementary. The total magnetic moment calculated is 2.414 μ_B_ with defect structure. Without producing defect, the moment is 0.0001 μ_B_ only. The defect produced moment is close to the magnetic moment calculated for other MoX_2_ compounds^18^. Interestingly, incorporation of defects in the crystal structure leads to ferromagnetic interaction from TDOS calculation which is consistent with those already reported^17,18,21^. The magnetism mainly promoted by Mo-*4d* orbital states.

## Conclusions

We presented a systematic study of magnetotransport and magnetic properties of single-crystalline MoTeP. The semiclassical two-band fitting of the Hall and longitudinal conductivity explain near-perfect carrier compensation at low temperature with very high carrier mobilities. It is evident from the Hall resistivity data that the transport properties in MoTeP are dominated by electron-type charge carriers. The suppressed magnetoresistivity is the result of reduced scattering effects due to the defect induced ferromagnetism. Particularly, at room temperature, this scattering is again suppressed due to the applied high magnetic field. At room temperature ρ_yx_ becomes nonlinear at higher field, implying that both type of carriers is activated. Below 25 K, the WAL-induced MR is extremely narrow within 1.5 T. Kohler’s scaling of MR% ~ (B/ρ_0_)^m^ with m = 1.65 together with a power law of B^n^ where n = 1.53 supports the dominating electron charge carrier transport. Departure from linearity above 50 K interprets the temperature dependent variation of electron and hole charge carriers. Finally, at room temperature electron and hole joint transport is observed from nonlinear S-shaped Hall data. Importantly, ferromagnetic nature from the asymmetric spin polarized total DOS near Fermi level supports our experimental observation of defect induced ferromagnetic MoTeP. We found incorporation of dopants into the system explored many intriguing features and open up another avenue for future material science research.

## Supplementary Information

Supplementary Information.
