# Supplementary material for: Defect induced ferromagnetic ordering and room temperature negative magnetoresistance in MoTeP
Source: Sci Rep. 2021 Apr 27;11:9104. doi: 10.1038/s41598-021-88669-8 (PMC8079386; doi:10.1038/s41598-021-88669-8)
Supplement: Supplementary file 1 — Supplementary Information. [file 41598_2021_88669_MOESM1_ESM.pdf]

Supplementary Material for

Defect induced ferromagnetic ordering and room temperature negative magnetoresistance in MoTeP

Debarati Pal[1], Shiv Kumar[2], Prashant Shahi[3], Sambhab Dan[1], Abhineet Verma[6], Vinod K. Gangwar[1], Mahima Singh[1], Sujoy Chakravarty[4], Yoshiya Uwatoko[5], Satyen Saha[6], Swapnil Patil[1*], Sandip Chatterjee[1*]

[1]*Department of Physics, Indian Institute of Technology (Banaras Hindu University), Varanasi-221005, India*
[2]*Hiroshima Synchrotron Radiation Center, Hiroshima University, Higashi-Hiroshima City, 739-0046, Japan*
[3]*Department of Physics, D.D.U. Gorakhpur University, Gorakhpur 273009*
[4]*UGC-DAE Consortium for Scientific Research, Kalpakkam Node, Kokilamedu, 603104, India*
[5]*Institute for Solid State Physics, University of Tokyo, Kashiwa, Chiba 277-8581, Japan*
[6]*Department of Chemistry, Institute of Science (Banaras Hindu University, Varanasi-221005, India*

1. The X-ray diffraction (XRD) pattern of the MoTeP material:

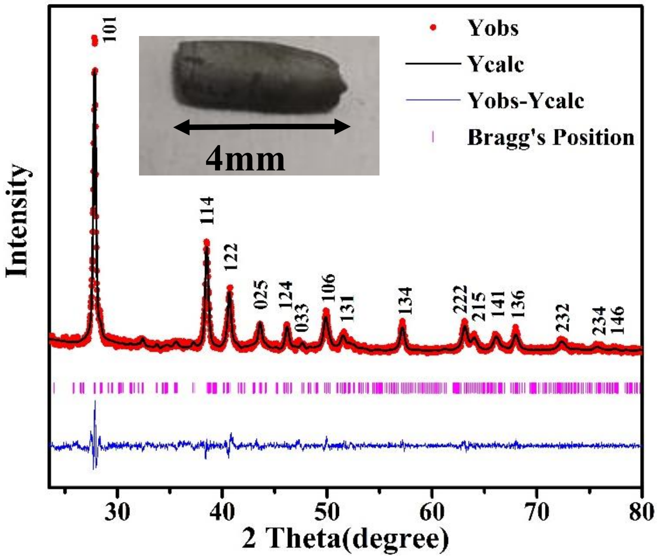

Fig. S1 XRD pattern of 1T′ MoTeP; inset: image of single crystal sample.

2. Temperature dependent Raman spectra:

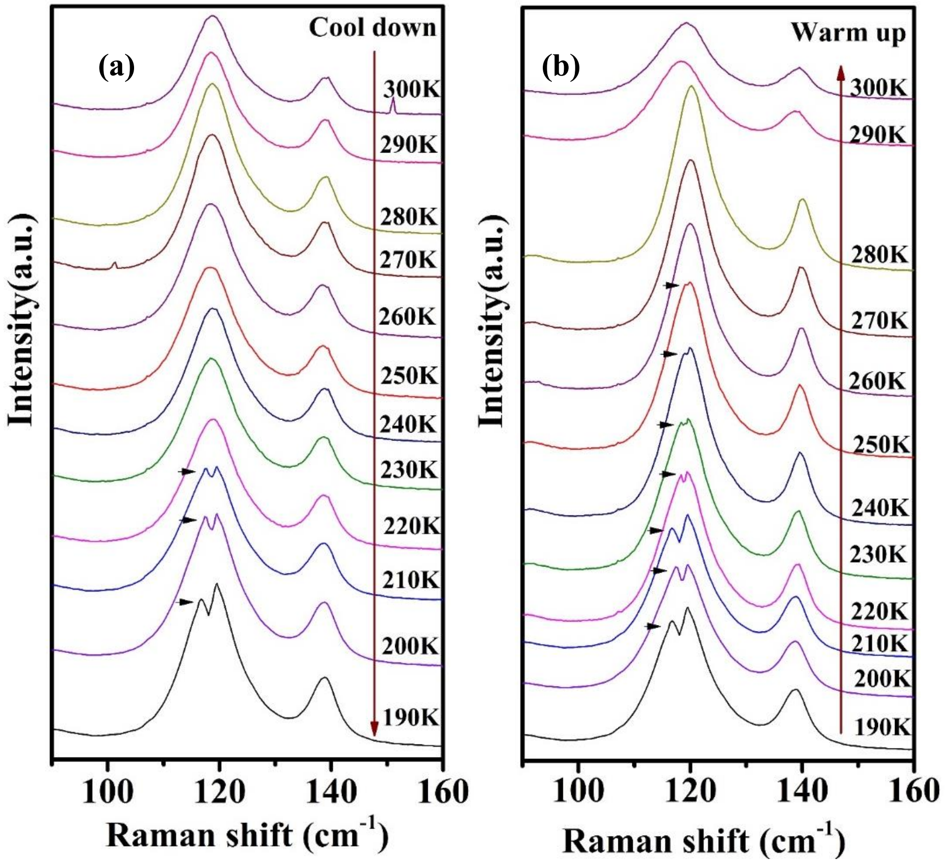

Fig. S2 Temperature dependent Raman spectra during (a) cooing and (b) heating cycle. The newly obtained mode is noted by arrow at 117.63 cm^{-1} indicating the presence of T_d phase.

3. Temperature evolution of the fitted intensity of newly Raman mode:

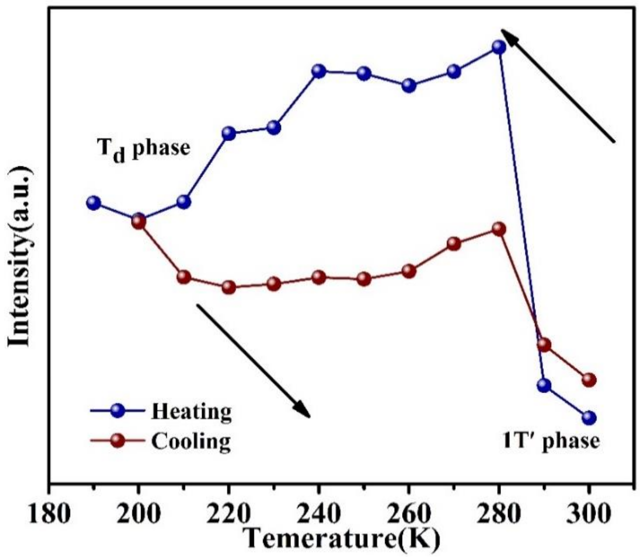

Fig. S3. Temperature evolution of the fitted intensity of newly Raman mode in two opposite temperature cycles.

4. Magnetic moment as a function of temperature in ZFC and FC cycle:

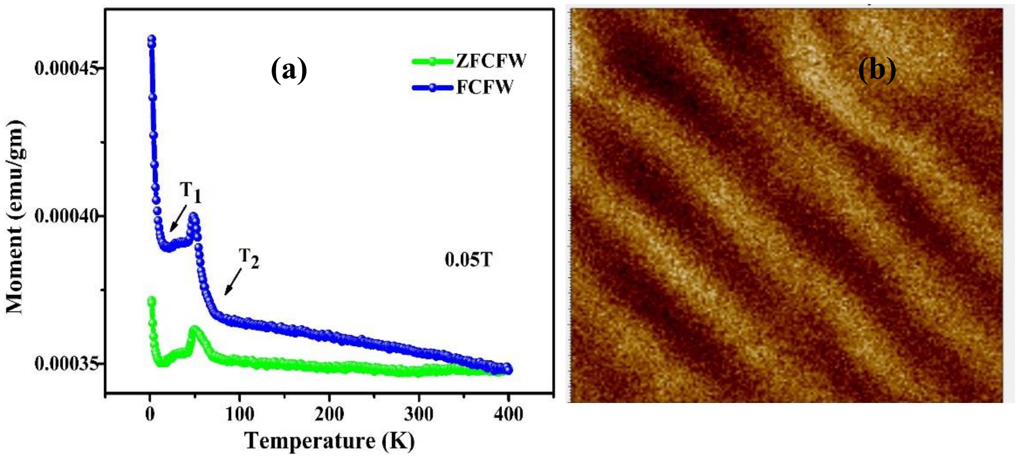

Fig. S4. (a) Magnetic moment as a function of temperature in ZFC and FC cycle; (b) MFM image taken at room temperature with scan areas 5×5 μm^2.

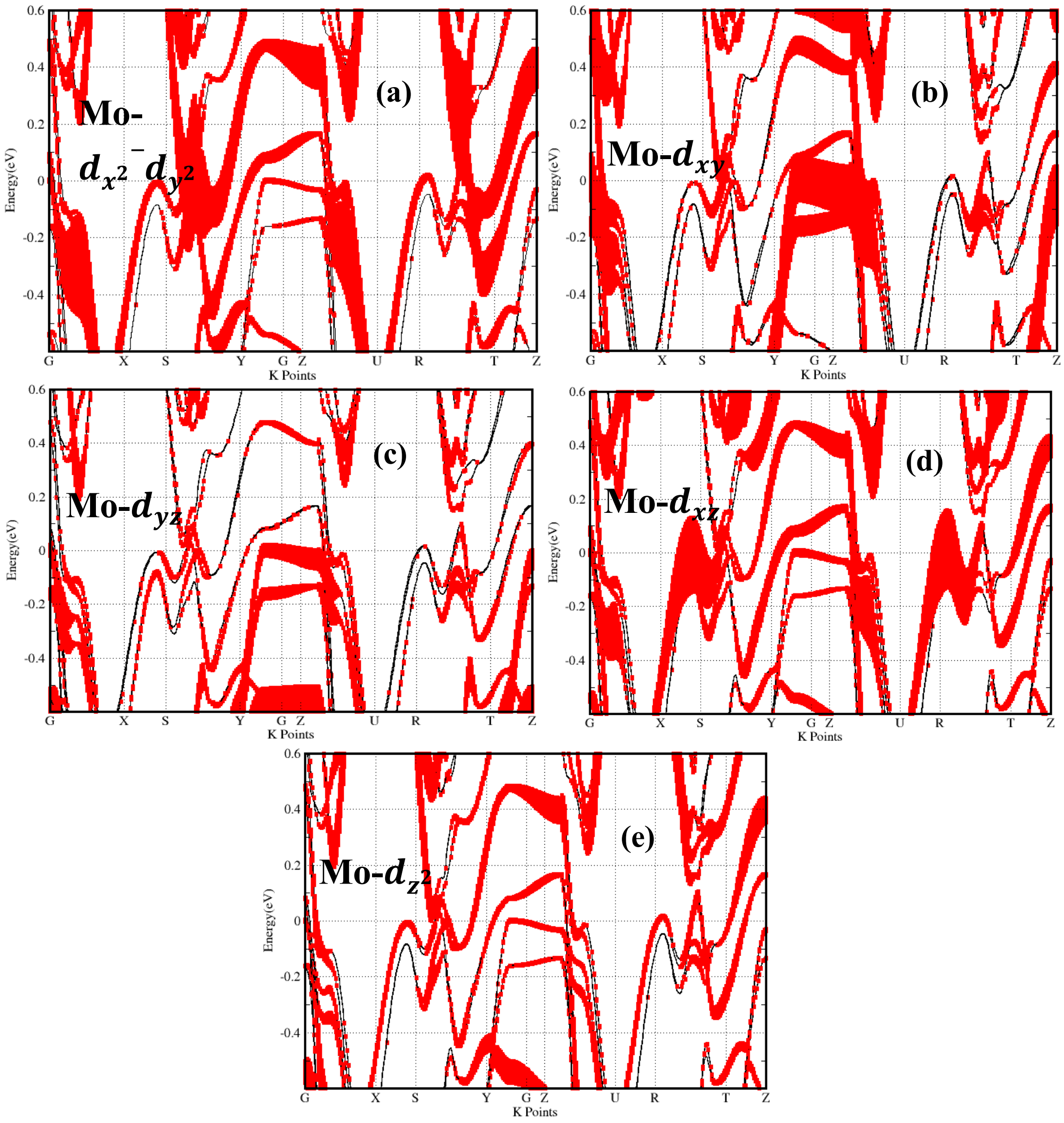

Fig. S5 Mo-d band contribution in bandstructure.

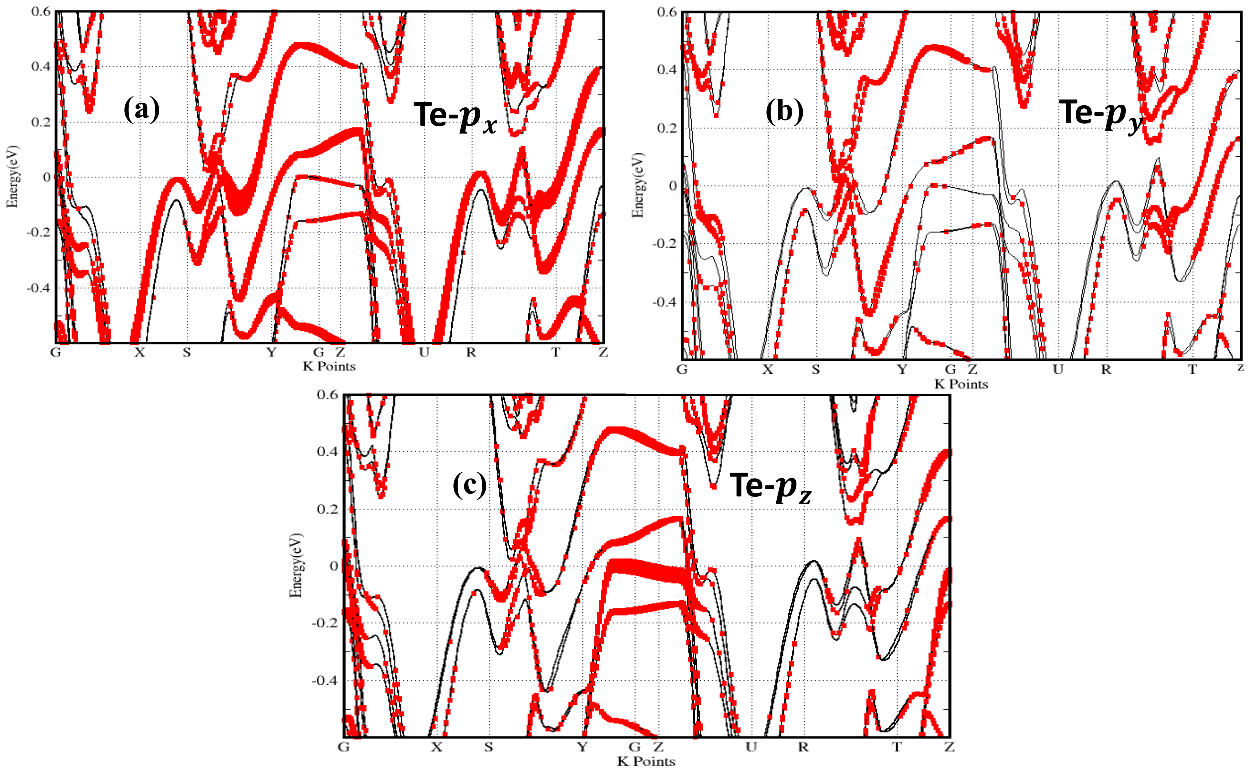

Fig. S6 Te-p band contribution in bandstructure.

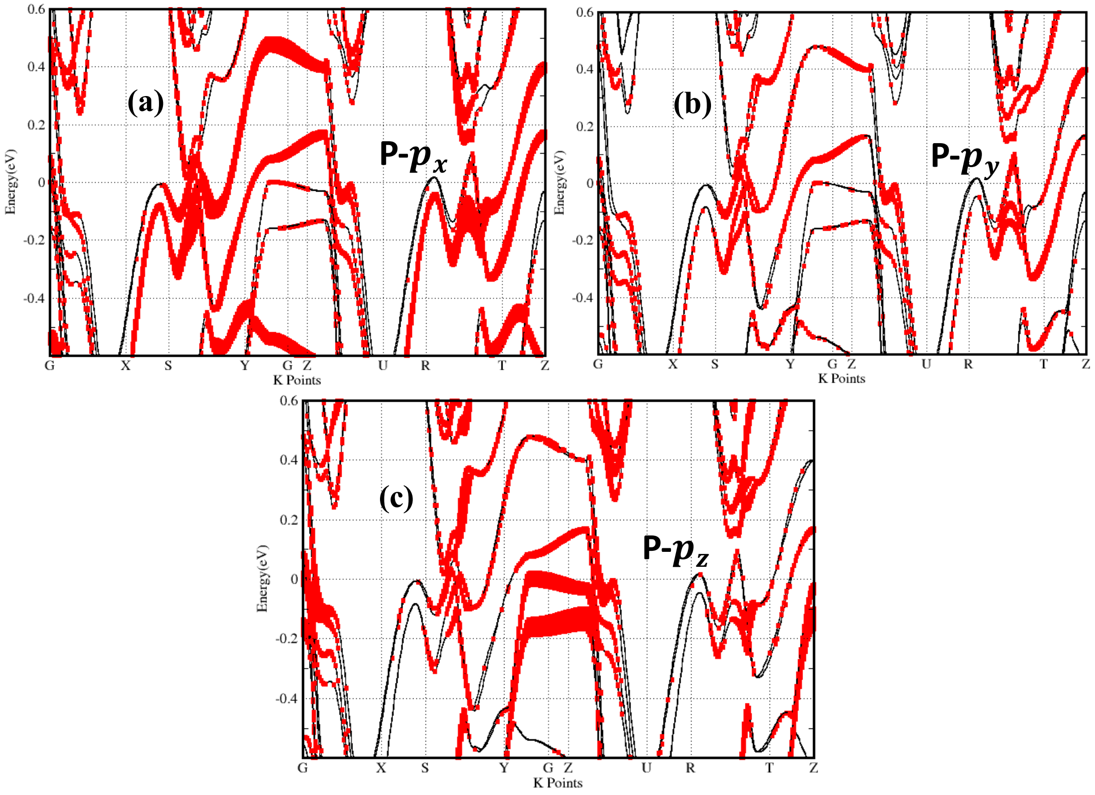

Fig. S7 P-p band contribution in bandstructure.

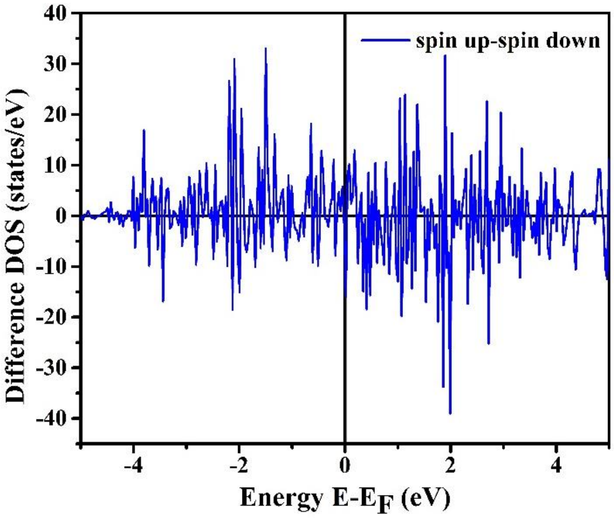

Fig. S8 Difference DOS of defect induced MoTeP.

We have demonstrated M-T behavior for the present investigation to probe the ferromagnetism into the sample. However, the data reveals a strong bifurcation between ZFC and FC cycle (figure S4a) which is might be due to ferromagnetic nature of the compound. This M-T behavior is in line with [1] defect produced $MoTe_2$. Long range magnetic ordering persists below the temperature T_1. The magnetic moment continuously increases below this point. Below temperature T_2, short range ordering (small ferromagnetic domains) is possibly present and responsible behind the inhomogeneous magnetism in the sample. This trend is similar to 2H-$MoTe_2$ and 2H-$MoSe_2$[1]. The bifurcation in the ZFC and FC response also suggests that different ferromagnetic domains tend to cancel out (anti-align) after ZFC. The domains will be aligned if we do field cooling.

We carried out Raman measurements to explore the origin of thermal hysteresis in both the heating and cooling path as depicted in figure S2a and S2b. We observed a single mode near 118.59 cm^{-1} to transform into two modes when sample was cooled down to ~220 K. A newly Raman mode at 117.63 cm^{-1} has been inspected as a signature of MoTeP-T_d phase. When the material is heated up to 250K the newly mode completely disappears. This signify the reappearance of 1T′ phase of MoTeP. The intensity behavior (figure S3) in the two routes are similar to $MoTe_2$ [2]. This is again indication of the existence of hysteresis in the material. Again, the undulating magnetic domains of the MFM image (figure S4b) is due to the ferromagnetic state, this is similar to the previous report on MoS_2[3].

We have shown d-band contribution of Mo in the bandstructure in figure S5a-e. The contribution of Te-p and P-p states are represented in figure S6 a-c and S7a-c. The contribution of Mo-d orbital is larger than Te-p and P-p states. The difference in spin up and spin down total DOS is illustrated in figure S8. The explains the presence of ferromagnetism in the material.

Reference-

1. Guguchia, Z. *et al.* Magnetism in semiconducting molybdenum dichalcogenides. *Sci. Adv.* **4**, eaat3672 (2018).
2. Yan, X. J. *et al.* Investigation on the phase-transition-induced hysteresis in the thermal transport along the c-axis of MoTe2. *npj Quantum Mater.* **2**, 1–7 (2017).
3. Han, S. W. *et al.* Electron beam-formed ferromagnetic defects on MoS2 surface along 1 T phase transition. *Sci. Rep.* **6**, 38730 (2016).

**Corresponding authors:* schatterji.app@iitbhu.ac.in
spatil.phy@itbhu.ac.in
